# Effectiveness of the Comprehensive-Humanized Care Model in nursing consultation: a case study with older adults

**DOI:** 10.1590/0034-7167-2025-0129

**Published:** 2025-11-21

**Authors:** Jessica Belen Rojas Espinoza, Beatriz Elizabeth Martínez Talavera, Daniela Mejía Medinal, María de los Ángeles Ponce Michua

**Affiliations:** I Universidad Autónoma del Estado de México. Toluca, Estado de México, Mexico

**Keywords:** Office Nursing, Primary Care Nursing, Aged, Healthcare Models, Case Reports., Enfermagem Ambulatorial, Enfermagem de Atenção Primária, Idoso, Modelos de Assistência à, Saúde, Relatos de Casos.

## Abstract

**Objectives:**

to determine the effectiveness of the Comprehensive-Humanized Care Model applied to primary care nursing consultations for older adults.

**Methods:**

a single-group, quasi-experimental, case study was conducted from 2023 to 2024 in the *Delegación Capultitlán*, Toluca, State of Mexico. The Nursing Care Process, comprehensive and geriatric assessment scales, and Kristen Swanson’s theory were used to implement nursing consultations.

**Results:**

through nursing interventions, positive changes were observed in older adults’ physical, functional, psychosocial, and spiritual well-being. Using Kristen Swanson’s Professional Care Scale, an average score of 58.3 was obtained, indicating that professional care is considered excellent.

**Final Considerations:**

the Comprehensive-Humanized Care Model is effective in maintaining older adults’ physical, functional, psychosocial, and spiritual well-being.

## RESUMO

**Objetivos:**

determinar a eficácia do Modelo de Cuidado Integral-Humanizado aplicado às consultas de enfermagem de atenção primária para idosos.

**Métodos:**

estudo de caso, quase-experimental, de grupo único, que foi conduzido entre 2023 e 2024 na *Delegación Capultitlán*, Toluca, Estado do México. O Processo de Cuidado de Enfermagem, as escalas de avaliação integral e geriátrica e a teoria de Kristen Swanson foram utilizados para implementar as consultas de enfermagem.

**Resultados:**

por meio das intervenções de enfermagem, foram observadas mudanças positivas no bem-estar físico, funcional, psicossocial e espiritual de idosos. Utilizando a Escala de Cuidado Profissional de Kristen Swanson, obteve-se uma pontuação média de 58,3, indicando que o cuidado profissional é considerado excelente.

**Considerações Finais:**

o Modelo de Cuidado Integral-Humanizado é eficaz na manutenção do bem-estar físico, funcional, psicossocial e espiritual de idosos.

## INTRODUCTION

In Mexico, as in other countries, life expectancy has increased and with it, the number of older adults who demand better social and health conditions to meet their specific needs, since the vast majority of them do not have healthy lifestyles or adequate self-care actions^(1)^. Therefore, nursing professionals must have specialized knowledge and skills to provide seniors with quality, effective, and humane care, without taboos or prejudices affecting the nurse-patient relationship^(2-4)^.

Nursing care must be comprehensive and personalized to ensure older adults’ well-being and quality of life^(5,6)^. Likewise, through health education and promotion, pillars of primary care, efforts must be directed toward implementing interventions that promote active and healthy aging, encourage self-care, reduce chronic degenerative diseases, and improve psychosocial and spiritual conditions that affect their health^(7,8)^.

In 2017, Kristen Swanson’s Caring Model was adapted to the care of non-institutionalized, functionally independent older adults attending an Adult Day Care Center in Toluca, Mexico. In-depth semi-structured interviews were conducted, from which six categories of care emerged, which were subsequently defined as therapeutic actions that intervene to achieve well-being: Family support; Hope; Movement; Health guidance; Therapeutic dialogue; and Love. In other words, in the five care processes of this theory (1) Maintaining belief; 2) Knowing; 3) Being with; 4) Doing for; and 5) Enabling), the elements that in older adults’ perception are required for nursing to provide comprehensive and humanized care were provided^(9)^.

Although there are efforts in Mexican healthcare institutions to provide comprehensive care to older adults, there is no nursing care model. Therefore, for this research, nursing consultation was used, understood as a care instrument that allows offering support to patients with their diagnosis, promotes clarification of doubts, guides on needs and facilitates patients’ therapeutic process^(10)^, in combination with the Comprehensive-Humanized Care Model for older adults (previously adapted from Kristen Swanson Care’s Caring Model for older adults) and the Nursing Care Process, to determine its effectiveness in older adult women’s well-being; in other words, the theoretical elements of care are put into practice and nursing interventions are assessed.

## OBJECTIVES

To determine the effectiveness of the Comprehensive-Humanized Care Model applied in nursing consultation in primary care for older adult women in the Delegación Capultitlán, Toluca, State of Mexico, through nursing interventions based on the scientific method and the study population’s real conditions, from February to July 2024, with the aim of promoting active and healthy aging.

## METHODS

### Ethical aspects

This study was approved by the Research Ethics Committee of the *Universidad Autónoma del Estado de México* School of Nursing and Midwifery. The bioethical principles of research, confidentiality and informed consent were also considered.

### Study design, period and place

A single-group, quasi-experimental case study was conducted during the 2023-2024 period in the Delegación de Capultitlán, Toluca, State of Mexico^(11)^.

### Information of older adults

Older adults over 60 and under 75 years of age with no alterations in their state of consciousness, functionally independent upon transfer, residing in Capultitlán, and who complied with attending their assessment and nursing interventions at least once a week in the Delegation’s multipurpose room were included in the study. Older adults who did not complete the nursing assessment phase were eliminated.

### Clinical findings

To assess older women’s functionality, the Katz Scale was administered to the 14 potential participants. Eight of them achieved a type A level of independence (independent in performing activities of daily living), and six achieved a type B score (independent in all their functions except one). Therefore, from this last group, three adults were discarded (but continued with the nursing intervention program) due to difficulty moving due to vision or musculoskeletal problems. The assessment phase was concluded with 11 older adults.

Moreover, an identification form was designed and used, which identified older adults as having a mean age of 67 years; 36.36% were widowed; 36.36% were married; 13.64% were single; and 13.64% were in a common-law relationship. Concerning family history, the diseases mentioned were diabetes mellitus, high blood pressure, arthritis, dementia, and a history of heart disease. Important history of surgery was the prevalence, with 81.81% related to cesarean sections, hysterectomies, and benign tumors; 36.36% related to falls without serious injuries; 18.18% related to sprains; 9.09% related to drug allergies (ketorolac); and 18.18% related to other important history (lung diseases, cancer, and hip injuries). Concerning older adults’ current health, 36.36% had high blood pressure and diabetes mellitus, respectively; 18.18% had arthritis; and other diseases (venous insufficiency, diabetic neuropathy, glaucoma, colitis, gastritis, and hypercholesterolemia) accounted for 9.09% each.

Diagnostic assessment was carried out using Marjory Gordon’s Functional Patterns Nursing Assessment, applied using a survey technique. Although Kristen Swanson’s Caring Model implicitly includes the five stages of the nursing process, there is still no instrument available to perform assessment based on her theory. Therefore, the assessment instrument applied does not contradict Swanson’s Caring Model; on the contrary, it helps in the identification of vital processes or real or potential health problems that favor the determination of nursing diagnoses and, therefore, promote knowledge of a person’s health status (knowledge). Specifically, for assessing older adults, the following patterns were considered, based on the previous study of 2017, where most of the problems found are accentuated:

“Health perception-health management”: regarding regular medical care, it was found that 54.54% of older women do not go to a health center; only 36.36% receive regular medical care; 63.63% receive appropriate medical follow-up; and 36.36% do not receive appropriate medical follow-up. “Nutritional-metabolic”: 54.54% of older adults have incomplete dentures; 27.27% have complete dentures; and 18.18% use dentures. Moreover, 63.63% of older women eat three meals a day; 18.18% eat two meals; 9.09% eat five meals with snacks; and 9.09% report eating only one meal a day. “Elimination”: 54.54% of older adults do not report any alterations in bowel elimination, while 27.27% have constipation and 18.18% have diarrhea; 18.18% report incontinence; and 9.09% have nocturia. “Sleep-rest”: 81.81% of older adults sleep between 5 and 7 hours, and of this percentage, 18.18% associate it primarily with insomnia and only 18.18% sleep more than 7 hours. “Cognitive-perceptual”: 54.54% of older adults report astigmatism, glaucoma, and decreased vision, while 45.45% have impaired vision and balance or declining hearing. “Role-relationship”: 63.63% of older adults report that their relationship with their family is good; 18.18% indicate that it is excellent; and 18.18% report that their relationship with their family is average. Furthermore, 36.36% of older adults live with their spouse on a daily basis; 36.36% live with their children; and 27.27% live with other people related to their work environment. “Coping-stress tolerance”: the activities they most like to relax with are manual activities, which account for 27.27% of the total, while intellectual activities account for 18.18%, sports activities account for 18.18%, and social activities account for 18.18%.

Based on the data obtained, nursing diagnoses were identified and care plans were developed considering the NANDA, NIC and NOC taxonomies.

### Timeline

At the end of January and beginning of February 2024, the call for participation was disseminated, inviting older adults from the Toluca Valley to be part of the study group, for which parks, avenues, churches were visited, and flyers were posted in visible places.

Since the above did not yield the expected response due to the lack of older adults at the agreed-upon location, it was decided to change the intervention site to the *Delegación de Capultitlán*, in Toluca, thereby increasing the likelihood of reaching out to older adults, given that it was a central area close to them. A request was made to the delegates, and they agreed to carry out the project in the multipurpose room. Therefore, outreach was restarted in the last two weeks of February and early March, inviting older adults to participate on Tuesdays, Wednesdays, and Thursdays, from 10:00 a.m. to 2:00 p.m.

The instruments were administered from March to May, depending on the age of each older adult. It began with the reading and signing of the Informed Consent Form and, subsequently, the Katz index was administered to determine whether participation was feasible with a level of independence of A or B. Instrument application had a minimum duration of two hours, having two to four sessions to complete the assessment and the unstructured interview, because through the questions that arose from the data of greatest interest, such as self-discovery and the relationship they had with others and with their environment, time for assessments was extended in most cases.

### Therapeutic intervention

Based on data obtained through comprehensive nursing assessment and geriatric assessment scales, older adults’ real and potential problems were determined using the NANDA 2020-2023 Taxonomy, which were used as a basis to designate the nursing outcomes and interventions considered in the planning stage.

These care plans were developed and implemented during nursing consultation provided for approximately six months to six older adults, who concluded the intervention phase, led by two professors and a team of four undergraduate nursing interns. At the same time as the comprehensive assessment was being conducted, some nursing interventions were implemented according to the needs identified during the survey.

**Figure 1:**
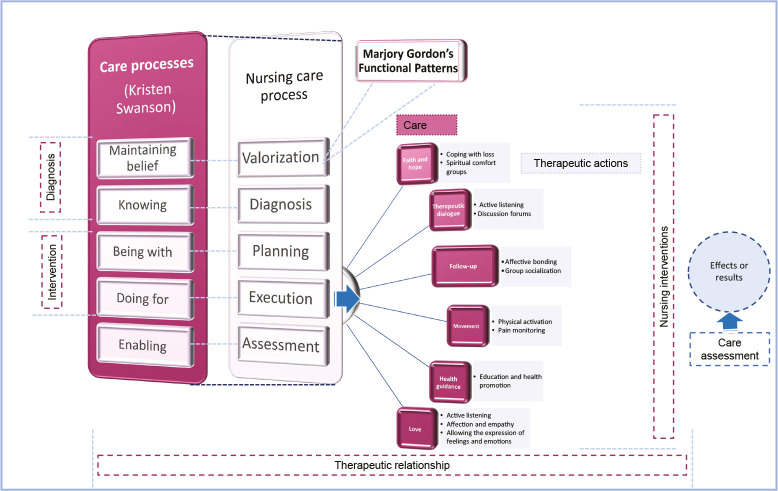
Comprehensive-Humanized Care Model for Older Adults, Toluca, State of Mexico, Mexico

In turn, nursing consultation took the Comprehensive-Humanized Care Model, derived from Kristen Swanson’s Theory, as a reference for the nurse-older adult relationship. The processes of being with and doing for correspond to the intervention phase, in which six nursing care approaches were taken as axes: 1) Faith and hope; 2) Therapeutic dialogue; 3) Follow-up; 4) Movement; 5) Health guidance; and 6) Love. Twelve therapeutic actions are derived, which are executed with the nursing interventions and activities (Figure 1).

It should be noted that this model emphasizes maintaining a therapeutic relationship that arises from trust and humanized care between nursing professionals and older adults, considering older adults at all times as “that person who is in the last stage of life, undergoing physical, psychological, social and spiritual changes, and who is characterized by being wise, expert, patient and tolerant, as a result of the transition associated with lived experience, acquired knowledge, losses and reflection on their lives”^(9)^. Likewise, care and therapeutic actions arose from the needs from the point of view of older adults, not nursing; in other words, they are specific to the perception and expectations of care of older women.

The interventions carried out with older adults are presented in the following charts, according to the spheres that make up the human being: physical-functional sphere (Chart 1); psycho-social sphere (Chart 2); and spiritual sphere (Chart 3).

**Chart 1:** Care, therapeutic actions, and nursing interventions in older adults’ physical and functional spheres, Toluca, State of Mexico, Mexico

Care proposed in the Comprehensive-Humanized Care Model	Therapeutic action proposed in the Comprehensive-Humanized Care Model	Nursing interventions (NIC)	Activities
Follow-up	Affective bonding	Facilitate self-responsibility	Self-awareness
		Improve sleep	Music therapy/aromatherapy
		Fall prevention	Physical activity/sensory stimulation
	Group socialization	Nutrition management	Healthy eating in a group
		Energy management	Open discussion
Health guidance	Health education	Help with self-care	Educational talks/self-care promotion
		Nutritional counseling	Educational talks
	Health follow-up	Urinary incontinence care	Pelvic floor exercises
		Vital sign monitoring	Measuring at the beginning of consultation
		Hyperglycemia management	Glucose tests/healthy diet
Faith and hope	Coping with loss	Mood control	Reading reflection
Movement	Physical activation	Exercise therapy: balance	Physical activity/recreational activities
		Exercise therapy: joint mobility	Physical activity/recreational activities
		Exercise promotion	Physical activity
	Pain follow-up	Pain management	Use of hot packs and pads

*Source: comprehensive assessment and nursing diagnoses in older adults in the Toluca Valley, 2024.*

**Chart 2:** Care, therapeutic actions, and nursing interventions in older adults’ psychosocial sphere, Toluca, State of Mexico, Mexico

Care proposed in the Comprehensive-Humanized Care Model	Therapeutic action proposed in the Comprehensive-Humanized Care Model	Nursing interventions (NIC)	Activities
Follow-up	Affective bonding	Emotional support	Readings
		Self-esteem boost	Open discussion
		Values clarification	Autobiography
		Group therapy	Recreational activities/guided tours
	Group socialization	Assertiveness training	Socializing
		Boost socialization	Board games
		Increase support systems	Fostering relationships with family and friends
		Behavior modification: social skills	Physical activity
Health guidance	Health education	Support for the primary caregiver	Educational talks
		Improve coping	Coloring/crafts
		Reduce anxiety	Relaxation and meditation activities
	Health follow-up	Boost self-awareness	Reflections on their lives
		Emotional support	Active listening
		Reality orientation	Physical activity
		Memory training	Memory games
		Cognitive stimulation	Memory games/board games
		Anxiety reduction	Music therapy/aromatherapy
Faith and hope	Coping with loss	Counseling	Educational talks
		Family therapy	Reflections on their lives
		Facilitate forgiveness	Cognitive map
		Support in decision-making	Open discussion
	Spiritual comfort group	Give hope	Reminiscence therapy
Therapeutic dialogue	Active listening	Family support	Word search
		Ease grief	Letter writing
		Promote family integrity	Building relationships with family and friends
	Discussion forums	Respite care	Music therapy
		Promote family involvement	Educational talks
Love	Allowing expression of feelings and emotions	Emotional support	Reminiscence therapy
		Mood management	Music therapy/aromatherapy
	Caring and empathy	Relaxation therapy	Coloring/crafts
		Improve self-confidence	Physical activity/board games

*Source: comprehensive assessment and nursing diagnoses in older adults in the Toluca Valley, 2024.*

**Chart 3:** Care, therapeutic actions, and nursing interventions in older adults’ spiritual sphere, Toluca, State of Mexico, Mexico

Care proposed in the Comprehensive-Humanized Care Model	Therapeutic action proposed in the Comprehensive-Humanized Care Model	Nursing interventions (NIC)	Activities
Faith and hope	Coping with loss	Spiritual support	Reflections on their lives
		Self-modification	Self-knowledge/autobiography
		Bibliotherapy	Reading poems or literary writings
		Provide hope	Incorporating elements of their culture
		Facilitate self-responsibility	Promoting self-care
		Presence	Acceptance, empathy, trust, and support
	Spiritual comfort groups	Facilitate religious practice	Prayers and thanksgiving
		Relaxation therapy	Aromatherapy
		Boost daily living skills	Healthy relationships
		Facilitate meditation	Simple guided imagery
		Spiritual support	Reflections on their lives
		Cultural mediation	Open discussion
Therapeutic dialogue	Discussion forums	Self-care assistance	Self-care
		Individual counseling	Educational talks
		Music therapy	Music therapy
		Decision-making support	Active listening
		Behavior modification	Self-awareness
	Active listening	Active listening	Active listening
		Cognitive restructuring	Simple guided imagery
		Simple guided imagery	Stories
		Spiritual support	Reflections on their lives
		Improve coping	Fostering relationships with family and friends
		Reduce anxiety	Relaxation therapies
		Provide hope	Incorporating elements of their culture
		Emotional support	Active listening
Follow-up	Affective bonding	Facilitate spiritual growth	Reflections on their lives
		Maintain processes	Open discussion
		Family support	Educational talks
	Group socialization	Counseling	Reflections on their lives
	Mutual support groups	Facilitate spiritual growth	Aromatherapy

*Source: comprehensive assessment and nursing diagnoses in older adults in the Toluca Valley, 2024.*

## RESULTS

Once this intervention phase was completed, the service was assessed in two stages: first, the assessment instruments were reapplied to determine the effects or outcomes on women’s health. The results were analyzed quantitatively, with preand post-tests of the geriatric assessment scales administered to older adults at the beginning and end of nursing consultation period.

Kristen Swanson’s Professional Care Scale was also used to assess older women’s perceptions of the nursing care they received. Finally, some comments from the unstructured interviews conducted during the intervention period are also presented.

### Pre and post-test of geriatric assessment

To complement comprehensive nursing assessment (based on functional patterns), validated geriatric assessment scales were applied in the human domains detailed above.

For the physical-functional domain, the Nutritional Assessment Scale was administered, yielding an average of 6.3 points in pre-test, which was interpreted as a high nutritional risk in older adults. Post-test averaged 4.8 points, interpreted as a moderate nutritional risk. It is worth mentioning that, among the interventions carried out, healthier food intake was promoted and encouraged.

Also in this area, the Tinetti Gait and Balance Assessment Tool was administered, and no significant changes were observed in post-intervention assessment, maintaining an average score between 24.6 and 25 points, which is interpreted as low risk.

Regarding the psychosocial sphere, the Family Apgar Questionnaire helped us verify the family functioning of the participating older adults, obtaining an average score of 12.3 in pre-test, which is interpreted as moderate dysfunction, and an average score of 14.3 in post-test, interpreted as mild dysfunction. It is worth highlighting that the nursing interventions focused on conflict resolution and forgiveness may have influenced older adults’ perception of family relationships.

The Social Resources Scale was used to assess the quality of older adults’ support networks. It was observed that each older adult had specific characteristics based on their social roles and those of their children and partners; however, all had deteriorating social resources. In pre-test, one older adult was identified as having severe social risk, two with moderate social impairment, and three with mild social impairment. In post-test, there were no significant changes, with one older adult at severe social risk, one with moderate social impairment, two with mild social impairment, and two with good social resources.

Using the Mini Mental State Examination, it was possible to identify that during the preand post-test survey periods older adults did not present evident signs of cognitive decline, maintaining an average of 29.8 points in the preand post-test and 32.1 in the post-test, which according to this scale is interpreted as within the normal range. There is a slight variation toward improvement, which could be due to the cognitive and memory activities that were worked on with older adults, including reading and writing.

Additionally, the Beck Anxiety Inventory was administered. At baseline, older adults had an average score of 6.8 points, indicating normal anxiety. However, in post-test, they obtained an average score of 13.8, indicating mild anxiety.

Using the Geriatric Depression Scale (Brink and Yesavage), it was again confirmed that older adult women who are functional and independent do not suffer from depression. The preand post-test averages ranged between 3.5 and 3.2 points, which is interpreted as a normal state.

Finally, for the spiritual sphere, the Trifactorial Spirituality Scale was applied to older Mexican adults. The women scored an average of 134 points at the beginning of the assessment and 138.6 points at the end of the intervention. This means that older adults have good spirituality.

### Kristen Swanson’s Professional Care Scale for nursing care

As part of nursing care assessment, it is important to understand older adults’ perceptions of the staff’s attitude, humanism, and values during nursing consultation. The average score obtained using Kristen Swanson’s Professional Care Scale was 58.3, which, according to the scale’s author, indicates that professional care is considered excellent. It involves being fully present, with loving healing flowing. Personality is mutually recognized; attention is negotiated; and professional intimacy develops. This relationship fosters spiritual freedom. This result was expected with the implementation of the Comprehensive-Humanized Care Model for older adults.

### Comments

During the open-ended interviews conducted in the nursing office, it was observed that older adults enjoyed company and being listened to, liked plants and their pets, with whom they would converse, and longed for peace and quiet. They were kind and engaged in activities, even sharing food and daily activities with the researchers. Below are some comments from the final session:

*It’s sad to know we won’t see them again.* (AM1)

*Thank you so much for so much love and affection. Congratulations on so many thoughtful gestures.* (AM2)

*What, are they just going to heal the external wounds? The internal wounds of these patients need to heal.* (AM3)

In addition, older adults kept a diary of experiences during the intervention period, highlighting the following fragments:

*My journey with nurses Laura, Gela, Dani, and Diego. To have a healthy phase of my later life, I’ve recounted my life from childhood to where I am now, and it’s at this stage that, at times, I’ve felt nostalgic. I think we should get used to living our own lives, to taking care of ourselves, because no one but ourselves knows what hurts us; we have to take care of ourselves while we can.* (AM1)

*My arrival at old age has been like a summer afternoon, a life of wonderful memories, experiences, reflections, of everything I was able to learn, know, recognize the great love of God, of all the wonderful things that he allowed me to have: my three children, that I must prepare for old age, to have a healthier life, to dedicate myself to myself, because as I no longer have a job, I need help to not get depressed, to give and dedicate myself to me, to know places, prepare myself for a calmer and healthier old age and get closer to God, who is always with me, and to be more independent every day.* (AM2)

## DISCUSSION

The results obtained demonstrate the effectiveness of the Comprehensive-Humanized Care Model in the areas that impact older adults’ health and well-being.

These results coincide with those of Yushan Yu^(12)^, who, through a systematic review, concluded that it is important to adapt home services to older adults’ needs, as well as to continue researching to provide better community and home services. In the physical and functional spheres, the health guidance strategy promoted self-care through healthy food consumption, which is consistent with the evidence presented in systematic reviews and documentary studies^(13,14)^. In addition, the group participation of older adults stands out, which marks a difference in the improvement presented in the results corresponding to this area and which are also related to what was presented in the systematic review by Teggart^(15)^, while emphasizing further research into older adults’ nutritional status.

The results obtained in the psycho-social sphere are in line with those found by Sánchez-Coronel^(16)^, who from the Mexican context recognizes that the lack of strategies in addressing the older population and family dynamics limits the field of action, which is addressed in the present investigation, showing how a set of structured interventions previously verified by the NIC taxonomy, which improved participants’ social and emotional dynamics. In this area, it is also demonstrated how the influence of group work, in conjunction with individualized interventions in conflict resolution and forgiveness, can improve family dynamics and spiritual self-care^(17)^. The data obtained show that the older adults who participated, by not having direct care within the family dynamic, join the need raised by the World Health Organization^(18)^ for age-friendly cities and communities, which promote healthy aging by optimizing health and safety resources.

In relation to cognitive aspects, the literature agrees that regular cognitive stimulation activities, such as those implemented in this study (reading, writing, memory games), can maintain and improve mental functions^(18-20)^. A finding of results is the increase in the level of anxiety from normal to mild, which is secondary to the application of the reminiscence intervention, although it is not clearly demonstrated as an effective intervention in anxiety, cognitive impairment and depression treatment^(21)^; at least documented, it produces the effect of science, recalling existing concerns. This fact suggests that further work is needed with these interventions to alleviate these feelings^(6)^ and promote a process of coping.

Given older adults’ health status and evident improvements shown in the results, the spiritual dimension and perception of the nursing care received are considered within the Comprehensive-Humanized Care Model as part of the basis of holistic health, since the spiritual state allows to enhance the benefits of the interventions described above. Therefore, Soto^(22)^, who makes an analysis building a state of the art based on the literature, has become an element of knowledge for nursing and specifically, confirming its usefulness in the care of older adults’ lives^(7,23)^.

These considerations allow for amalgamation of a series of individualized interventions, which is reflected in professional care assessment, validating the effectiveness of the Comprehensive-Humanized Care Model. Furthermore, such satisfaction is considered to be linked to home care, as reported by studies from Brazil and Spain^(24-27)^, which relate satisfaction with care to personalized and holistic care.

The effectiveness of spiritual interventions is reflected in testimonials such as “developing and maintaining at advanced ages the functional capacity that makes well-being possible”, which demonstrate a connection between spiritual well-being and functionality.

### Study limitations

It can be considered that the sample was small due to the difficulty in integrating a group of older adults who met the study requirements and who, above all, had the availability and time to attend nursing consultation, since, in Mexico, the majority of older women play the role of caregivers for their grandchildren and the home, making it difficult for older adults to attend activities or interventions for their self-care.

### Contributions to nursing

Among the specific contributions of the Comprehensive-Humanized Care Model is its application to nursing consultation in the geriatric area, demonstrating the autonomy and efficiency of spiritually based care and interventions, as well as improvements in the physical and psychosocial spheres. Furthermore, the scientific evidence and integration of Kristen Swanson’s theory with community nursing practice are expanded.

## FINAL CONSIDERATIONS

The results of nursing interventions were positive for older adults’ well-being. Regarding functionality, through the exercises and physical activity implemented as part of the interventions, women experienced less pain in their joints and increased strength in their legs. Psychosocially, as older adults’ trust was gained, they verbalized some of their family members’ problems or attitudes that made them feel unwell. However, it was noticeable that they were happier to participate in the activities proposed by the research group, and bonds of friendship were forged among older adults, building their own support network. It allowed the expression of spiritual thoughts and activities, which brought them greater tranquility and peace in their lives.

It is suggested to replicate the methodology and model in larger populations of older adults to compare the results with those of this study.

## Data Availability

The research data are available in a repository: https://doi.org/10.48331/scielodata.JHVJAT.

## References

[1] Rebolledo MD, Vargas AG, Oviedo RR, Quijije DR. (2021). Perspectiva del cuidado cotidiano del adulto mayor: un estudio fenomenológico y social. Conecta Libertad.

[2] Bustamante ELK, Ochoa MKM, Yamasqui PJI, Rodríguez PPE. (2023). Atención en el cuidado del adulto mayor residente en centros gerontológicos: una revisión sistemática. Proscien.

[3] Placencia LBM, Mendoza MCE. (2024). Percepción de los adultos mayores sobre comportamientos del cuidado de enfermería humanizado, Hospital de Especialidades Portoviejo.

[4] Saucedo ERV. (2024Abr). Calidad de atención de enfermería en el adulto mayor hospitalizado: revisión sistemática. Vive Rev Salud.

[5] Arroyo CFJ, Morales RMC. (2024Abr). Importancia de la atención integral de enfermería en adultos mayores residentes de establecimientos para ancianos. rhe.

[6] Ramírez JGM, Hernández EAT, Guerrero-Castañeda RF. (2023). Implementation of a nursing strategy for self-transcendence in older adults: an experience report. Rev Bras Enferm.

[7] Guerrero CRF, Acevedo LMN, Reyes MBR. (2023). Cuidado humano al adulto mayor en la comunidad para favorecer un envejecimiento saludable. CIETNA.

[8] Rodrigues RAP, Fhon JRS, Lima FM. (2021). El Cuidado del adulto mayor en la atención primaria en salud em tiempos de COVID-19.

[9] Rojas EJB, García HMD, Cárdenas BL, Vázquez GL, Silveira KS. (2018). Adaptación del modelo de Kristen Swanson para el cuidado de enfermería en adultas mayores. Texto Contexto Enferm.

[10] Macêdo SM, Sena MCS, Miranda KCL. (2013). Consulta de enfermagem ao paciente com HIV: perspectivas e desafios sob a ótica de enfermeiros. Rev Bras Enferm.

[11] Gagnier JJ, Kienle G, Altman DG, Moher D, Sox H, Riley D, the CARE Group (2013). The CARE Guidelines: Consensus-based Clinical Case Reporting Guideline Development.

[12] Yushan Y, Jun Z, Mirko P, Xudong Z, Wei-Hong Z. (2024). Utilization of homeand community-based services among older adults worldwide: a systematic review and meta-analysis. Int J Nurs Stud.

[13] Dent E, Wright ORL, Woo J, Hoogendijk E. (2023). Malnutrition in older adults. Lancet.

[14] Azzolino D, Lucchi T. (2023). Malnutrition in older adults: a wider view. Lancet.

[15] Teggart K, Ganann R, Sihota D, Moore C, Keller B, Senson C, Phillips SM, Neil-Sztramko S (2022). Group-based nutrition interventions to promote healthy eating and mobility in community-dwelling older adults: a systematic review. Public Health Nutrition.

[16] Sánchez-Coronel G, Soto-Ávila V, Ramos-Durán JD, Ruiz Domínguez AE, Ramírez Aquino A. (2025). Asociación entre funcionalidade familiar y la sobrecarga del cuidado del adulto mayor. Cien Lat Rev Cien Multidisc.

[17] Le Blanc RG, Chiodo L, Jacelon CS. (2022). (Influencia de las relaciones sociales en el autocuidado y la salud de las personas mayores que viven con enfermedades crónicas: un estudio de métodos mixtos. Revista internacional de enfermería para personas mayores.

[18] Organización Mundial de la Salud (OMS) (2024). Metodología del proyecto de ciudades amigables con los adultos mayores de la OMS: Protocolo de Vancouver.

[19] Helbling M, Grandjean ML, Srinivasan M. (2023). Effects of multisensory environment/stimulation therapy on adults with cognitive impairment and/or special needs: a systematic review and meta-analysis. Special Care in Dentistry.

[20] Gómez-Soria I, Iguacel I, Aguilar-Latorre A, Peralta-Marrupe P, Latorre E, Cuenca Zaldívar JN (2023). Cognitive stimulation and cognitive results in older adults: a systematic review and meta-analysis. Arch Gerontol Geriatr.

[21] Gómez-Soria I, Marín-Puyalto J, Peralta-Marrupe P, Latorre E, Calatayud E. (2022). Effects of multi-component non-pharmacological interventions on cognition in participants with mild cognitive impairment: a systematic review and meta-analysis. Arch Gerontol Geriatr.

[22] Noboa Pullaguari KD (2023). La terapia de reminiscencia como coadyuvante en el deterioro cognitivo del adulto mayor. Rev Lat Cien Soc.

[23] Soto-Morales AM, Olivella-Fernández MC, Bastidas-Sánchez CV. (2020). Cuidado Espiritual al adulto mayor, elemento del conocimiento y la práctica de Enfermería. Rev Cien Cuid.

[24] Beltrán-Solórzano JM, Morillas-Bulnes AM. (2023). Cuidado espiritual de enfermería, una aproximación sistemática al estado de arte. Rev. Lat. Ciec. Sociales.

[25] Rodrigues MA, Santana RF, Hércules AB, Bela JC, Rodrigues JN. (2021). Teleenfermería en el Servicio de Atención Domiciliaria en la pandemia de COVID-19: un estudio transversal. Braz J Nurs.

[26] Nava LF, Duarte TTP, Lima WL, Magro MCS. (2022). Monitorización avanzada de enfermería: pacientes de riesgo en atención primaria. Esc Anna Nery.

[27] Hakimjavadi R, Levi C, LeBlanc K, Guglani S, Helmer-Smith M, Joschko J (2022). Consulta electrónica por parte de enfermeras de práctica avanzada para mejorar el acceso a atención especializada para adultos mayores. Rev Enferm Gerontol.

