# Supplementary material for: Effectiveness of the Comprehensive-Humanized Care Model in nursing consultation: a case study with older adults
Source: Rev Bras Enferm. 2025 Nov 21;78(Suppl 2):e20250129. doi: 10.1590/0034-7167-2025-0129 (PMC12659912; doi:10.1590/0034-7167-2025-0129)
Supplement: Translation

# Translation: Efectividad del Modelo de Cuidado Integral-humanizado en la consulta de enfermería: estudio de caso con adultas mayores

**DOI:** 10.1590/0034-7167-2025-0129es

**Authors:** Jessica Belen Rojas Espinoza, Beatriz Elizabeth Martínez Talavera, Daniela Mejía Medinal, María de los Ángeles Ponce Michua

**Affiliations:** I Universidad Autónoma del Estado de México. Toluca, Estado de México, México

**Keywords:** Enfermería de Consulta, Enfermería de Atención Primaria, Anciano, Modelos de Atención de Salud, Informes de Casos.

## Abstract

**Objetivos:**

determinar la efectividad del Modelo de Cuidado Integral-humanizado aplicado en la consulta de enfermería en atención primaria para mujeres adultas mayores.

**Métodos:**

estudio cuasiexperimental de un solo grupo, de tipo estudio de caso, realizado durante el periodo 2023-2024 en la Delegación de Capultitlán, Toluca, Estado de México. Para implementar la consulta de enfermería, fueron utilizados el Proceso Atención Enfermería, escalas de valoración integral y geriátricas que se aplicaron con un pre y *post-test*, así como la teoría de Kristen Swanson.

**Resultados:**

mediante las intervenciones de enfermería, se observaron cambios positivos en las esferas físico-funcional, psico-social y espiritual de las mujeres mayores. Con la Escala de Cuidado Profesional de Kristen Swanson, se obtuvo una puntuación promedio de 58.3, lo cual indica que el cuidado profesional es considerado excelente.

**Consideraciones Finales:**

el Modelo de Cuidado Integral-humanizado es efectivo para mantener el bienestar físico-funcional, psicosocial y espiritual de adultas mayores.

## INTRODUCCIÓN

En México, como en otros países, ha aumentado la esperanza de vida y con ello, la cantidad de adultos mayores que demandan mejores condiciones sociales y de salud para satisfacer sus necesidades específicas, ya que, en su gran mayoría, no tienen estilos de vida saludable ni acciones adecuadas de autocuidado^(1)^. Por tanto, los profesionales de enfermería deben contar con conocimientos especializados y competencias para brindar a los adultos mayores cuidados con calidad, efectividad y humanismo, sin que los tabúes o prejuicios afecten la relación enfermera-paciente^(2-4)^.

La atención de enfermería debe ser integral y personalizada, para garantizar el bienestar y la calidad de vida de los adultos mayores^(5,6)^. Asimismo, desde la educación y promoción para la salud, pilares del primer nivel de atención, se deben dirigir esfuerzos en la implementación de intervenciones que favorezcan el envejecimiento activo y saludable, fomentar el autocuidado, la disminución de enfermedades crónico-degenerativas, y mejorar las condiciones psicosociales y espirituales que afecten su salud^(7,8)^.

En 2017, se adaptó el Modelo de Cuidado de Kristen Swanson en la atención de adultas mayores no institucionalizadas y con independencia funcional que asistían a una Casa de Día en Toluca, México, para lo cual se aplicaron entrevistas semiestructuradas a profundidad, de las que emergieron seis categorías de cuidado que, posteriormente, se definieron como acciones terapéuticas que intervienen para el logro del bienestar: El acompañamiento familiar; La esperanza; El movimiento; La orientación en salud; El diálogo terapéutico; y El amor. Es decir, en los cinco procesos de cuidado de esta teoría (1) Mantener las creencias; 2) El conocer; 3) El estar con; 4) El hacer por; y 5) El permitir o posibilitar), se aportaron los elementos, que bajo la percepción de las adultas mayores, se requieren para que enfermería brinde un cuidado integral y humanizado^(9)^.

Aunque existen esfuerzos en las instituciones de salud de México para brindar atención integral al adulto mayor, no se cuenta con un modelo de cuidado de enfermería. Por lo tanto, para esta investigación, se empleó la consulta de enfermería, entendida como un instrumento asistencial que permite ofrecer apoyo al paciente ante su diagnóstico, promueve el aclaramiento de dudas, orienta sobre necesidades y facilita el proceso terapéutico de los pacientes^(10)^, en combinación con el Modelo de Cuidado Integral-humanizado para adultas mayores (antes adaptación del Modelo de Cuidado de Kristen Swanson en la atención de adultas mayores) y el Proceso Atención de Enfermería, para determinar su efectividad en el bienestar de mujeres adultas mayores; es decir, se ponen en práctica los elementos teóricos del cuidado y se evalúan las intervenciones de enfermería.

## OBJETIVOS

Determinar la efectividad del Modelo de Cuidado Integral-humanizado aplicado en la consulta de enfermería en atención primaria para mujeres adultas mayores de una Delegación de Toluca, Estado de México, mediante intervenciones de enfermería fundamentadas en el método científico y en las condiciones reales de la población de estudio, durante el periodo de febrero a julio de 2024, con la finalidad de propiciar un envejecimiento activo y saludable.

## MÉTODOS

### Aspectos éticos

Este estudio fue aprobado por el Comité de Ética en Investigación de la Facultad de Enfermería y Obstetricia de la Universidad Autónoma del Estado de México, así como también se consideraron los principios bioéticos de la investigación, la confidencialidad y el consentimiento informado.

### Diseño, periodo y localización del estudio

Estudio cuasiexperimental de un solo grupo, de tipo estudio de caso, realizado durante el periodo 2023-2024 en la Delegación de Capultitlán, Toluca, Estado de México^(11)^.

### Información de las adultas mayores

Se incluyeron en el estudio adultas mayores de más de 60 años y menores de 75 años sin alteraciones en su estado de conciencia, funcionalmente independientes en el traslado, con residencia en Capultitlán y que cumplieran con la asistencia a su valoración e intervenciones de enfermería, al menos una vez a la semana, en el salón de usos múltiples de la Delegación. Se eliminaron adultas mayores que no completaron la fase de valoración de enfermería.

### Hallazgos clínicos

Para evaluar la funcionalidad de las mujeres mayores, se aplicó la Escala de Katz a las 14 posibles participantes. Ocho de ellas obtuvieron un nivel de independencia tipo A (independientes para realizar las actividades de la vida diaria), y seis obtuvieron una puntuación de tipo B (independientes en todas sus funciones con excepción de una). Por lo tanto, de este último grupo se descartaron tres adultas (pero continuaron con el programa de intervenciones de enfermería), por presentar dificultad para trasladarse debido a problemas en la visión o músculo-esqueléticos, concluyéndose la fase de evaluación con 11 adultas mayores.

Además, se diseñó y utilizó una Ficha de Identificación, mediante la cual se distinguió que las adultas mayores se encontraban en una media de edad de 67 años; el 36.36% eran viudas; el 36.36% eran casadas; el 13.64% eran solteras; y el 13.64% estaban en unión libre. Respecto de los antecedentes heredofamiliares, las enfermedades mencionadas fueron diabetes mellitus, hipertensión arterial, artritis, demencia y antecedente de enfermedad cardiaca. Los antecedentes de importancia fueron la prevalencia de cirugías, con 81.81% relacionadas con cesáreas, histerectomías y tumores benignos; 36.36% relacionadas con caídas sin lesiones de gravedad; 18.18% relacionadas con esguince; 9.09% relacionadas con alergia a medicamentos (ketorolaco); y 18.18% relacionadas con otros antecedentes de importancia (enfermedades pulmonares, cáncer y lesiones de cadera). En lo referente a la salud actual de las adultas mayores, 36.36% presentan hipertensión arterial y diabetes mellitus respectivamente; 18.18% presentan artritis; y demás enfermedades (insuficiencia venosa, neuropatía diabética, glaucoma, colitis, gastritis e hipercolesterolemia) tienen un 9.09% cada una.

La evaluación diagnóstica se llevó a cabo a través del Instrumento de Valoración de Enfermería por Patrones Funcionales de Marjory Gordon, aplicado mediante la técnica de encuesta, ya que, si bien, el Modelo de Kristen Swanson tiene implícitas las cinco etapas del proceso de enfermería, aún no se cuenta con un instrumento para realizar la valoración con base en su teoría. Por lo que, el instrumento de valoración aplicado, no se contradice con el Modelo de Swanson; al contrario, coadyuva en la identificación de procesos vitales o problemas de salud reales o potenciales que favorecen la determinación de diagnósticos de enfermería y que, por lo tanto, propician el conocimiento del estado de salud de la persona (conocer). Específicamente, para la valoración de adultas mayores, se consideraron los siguientes patrones, con base en el estudio previo de 2017, en donde se acentúan la mayoría de problemas encontrados:

“Percepción-manejo de la salud”: en relación a la asistencia periódica al médico, se identificó que el 54.54% de las mujeres mayores no acuden al centro de salud; sólo 36.36% tiene asistencia médica periódica; el 63.63% llevan el seguimiento médico correspondiente; y el 36.36% no llevan el seguimiento médico correspondiente. “Nutricional-metabólico”: el 54.54% de las adultas mayores tienen dentadura incompleta; el 27.27% tienen dentadura completa; y el 18.18% usan prótesis. El 63.63% de las mujeres mayores realizan tres comidas al día; el 18.18% hacen dos comidas; el 9.09% hacen cinco con las colaciones; y el 9.09% indican que solo realiza una comida al día. “Eliminación”: el 54.54% de las adultas mayores no presentan ninguna alteración de la eliminación intestinal, mientras que 27.27% poseen estreñimiento y el 18.18%, diarrea; el 18.18% refieren incontinencia; y el 9.09% poseen nicturia. “Sueño-descanso”: el 81.81% de adultas mayores duermen entre 5 y 7 horas, y de este porcentaje, el 18.18% lo asocian principalmente al insomnio y solo el 18.18% duermen más de 7 horas. “Cognitivo-perceptual”: el 54.54% de las adultas mayores refieren astigmatismo, glaucoma y disminución en la vista, mientras que el 45.45% poseen alteración de la vista y el equilibrio o la audición que va disminuyendo. “Rol-relaciones”: el 63.63% de las adultas mayores indican que la relación con su familia es buena; el 18.18% indican que es excelente; y el 18.18% señalan que la relación con ellos es regular. El 36.36% de las adultas mayores conviven a diario con su cónyuge; el 36.36% conviven con sus hijos; y el 27.27% conviven con otras personas relacionadas a su entorno laboral. “Adaptación-tolerancia al estrés”: las actividades con las que más les gusta relajarse son las manuales, las cuales representan un 27.27% del total, mientras que las intelectuales, un 18.18%, deportivas, un 18.18% y de convivencia social también, un 18.18%.

Con base en los datos obtenidos, se identificaron los diagnósticos de enfermería y se elaboraron los planes de cuidado considerando las taxonomías NANDA, NIC y NOC.

### Línea de tiempo

A finales de enero e inicios de febrero de 2024, se realizó la difusión de la convocatoria de participación, con la que se invitaba a las adultas mayores del Valle de Toluca a ser parte del grupo de estudio, para lo cual se visitaron parques, avenidas, iglesias, y se pegaron *flyers* en lugares visibles.

Dado que, con lo anterior, no se obtuvo la respuesta esperada, porque no hubo asistencia de las adultas mayores en el punto acordado, se decidió cambiar el sitio de intervención a la Delegación de Capultitlán, Toluca, con lo cual aumentaban las probabilidades de un acercamiento con adultas mayores, puesto que era una zona central y cercana a ellas. Se realizó la solicitud a los delegados, y fue aceptado realizar el proyecto en el Salón de Usos Múltiples. Por lo que, se reinició la difusión en las últimas dos semanas de febrero e inicios de marzo, invitando a las adultas mayores los días martes, miércoles y jueves, de 10:00 a 14:00 horas.

La aplicación de los instrumentos se llevó a cabo durante los meses de marzo a mayo, de acuerdo al tiempo que poseía cada adulta mayor. Se inició con la lectura y firma del consentimiento informado y, posteriormente, con la aplicación del índice de Katz para verificar si era viable la participación con un nivel de independencia A o B. La aplicación de los instrumentos tenía una duración mínima de dos horas, teniendo de dos a cuatro sesiones para completar la valoración y la entrevista no estructurada, porque a través de las preguntas que surgieron de los datos de mayor interés, como el autodescubrimiento, la relación que tenían con los demás, con su entorno, el tiempo para las evaluaciones se extendió en la mayoría de los casos.

### Intervención terapéutica

Con base en los datos obtenidos mediante la valoración integral de enfermería y las escalas de valoración geriátrica, se determinaron los problemas reales y potenciales de las adultas mayores utilizando la Taxonomía NANDA 2020-2023, los cuales se tomaron de base para designar los resultados e intervenciones de enfermería considerados en la etapa de planificación.

Estos planes de cuidado se desarrollaron y ejecutaron durante la consulta de enfermería que se brindó durante aproximadamente seis meses a seis adultas mayores que concluyeron con la fase de intervención, a cargo de dos profesoras y un equipo de cuatro pasantes de pregrado en enfermería. Al mismo tiempo que se realizaba la valoración integral, se ejecutaron algunas intervenciones de enfermería de acuerdo a las necesidades que se iban identificando durante la encuesta.

A su vez, la consulta de enfermería tomó como referente en la relación enfermera-adulta mayor el Modelo de Cuidado Integral-humanizado, que deriva de la Teoría de Kristen Swanson. Los procesos estar con y hacer por corresponden a la fase de intervención, en la que se tomaron como eje seis cuidados de enfermería: 1) Fe y esperanza; 2) Diálogo terapéutico; 3) Acompañamiento; 4) Movimiento; 5) Orientación de salud; y 6) Amor y, se desprenden 12 acciones terapéuticas, mismas que se ejecutan con las intervenciones y actividades de enfermería (Figura 1).

**Figura 1:** Modelo de Cuidado Integral-Humanizado para adultas mayores, Toluca, Estado de México, México

Cabe decir que, con este modelo, se hace énfasis en mantener una relación terapéutica que surge de la confianza y el cuidado humanizado entre el profesional de enfermería y la adulta mayor, considerando en todo momento a la adulta mayor como “Aquella persona que se encuentra en la última etapa de la vida, cursando por cambios físicos, psicológicos, sociales y espirituales, y que se caracteriza por ser sabia, experta, paciente y tolerante, como resultado de la transición asociada a la experiencia vivida, el conocimiento adquirido, las pérdidas y la reflexión de su vida”^(9)^. Asimismo, los cuidados y acciones terapéuticas surgieron de las necesidades desde el punto de vista de las adultas mayores, no de enfermería; es decir, son específicos a la percepción y expectativas de cuidado de las mujeres mayores.

Las intervenciones realizadas con las adultas mayores se presentan en los siguientes cuadros, de acuerdo a las esferas que integran al ser humano: esfera físico-funcional (Cuadro 1); esfera psico-social (Cuadro 2); y esfera espiritual (Cuadro 3).

**Cuadro 1:** Cuidados, acciones terapéuticas e intervenciones de enfermería en la esfera físico-funcional de las adultas mayores, Toluca, Estado de México, México

Cuidados propuestos en el Modelo de Cuidado Integral-humanizado	Acción terapéutica propuesta en el Modelo de Cuidado Integral-humanizado	Intervenciones de enfermería (NIC)	Actividades
Acompañamiento	Vinculación afectiva	Facilitar la autorresponsabilidad	Autoconocimiento
		Mejorar el sueño	Musicoterapia/aromaterapia
		Prevención de caídas	Actividad física/estimulación sensorial
	Socialización grupal	Manejo de la nutrición	Alimentación saludable en grupo
		Manejo de la energía	Discusión abierta
Orientación de salud	Educación para la salud	Ayuda con el autocuidado	Charlas educativas/fomento del autocuidado
		Asesoramiento nutricional	Charlas educativas
	Seguimiento de la salud	Cuidados de la incontinencia urinaria	Ejercicios de suelo pélvico
		Monitorización de signos vitales	Medición al inicio de la consulta
		Manejo de la hiperglucemia	Glucometrías/alimentación saludable
Fe y esperanza	Afrontamiento de pérdidas	Control del estado de ánimo	Reflexión de lectura
Movimiento	Activación física	Terapia de ejercicios: equilibrio	Actividad física/actividades recreativas
		Terapia de ejercicios: movilidad articular	Actividad física/actividades recreativas
		Fomento del ejercicio	Actividad física
	Seguimiento del dolor	Manejo del dolor	Uso de compresas calientes y almohadillas

*Fuente: valoración integral y diagnósticos de enfermería en adultas mayores del Valle de Toluca, 2024.*

**Cuadro 2:** Cuidados, acciones terapéuticas e intervenciones de enfermería en la esfera psico-social de las adultas mayores, Toluca, Estado de México, México

Cuidados propuestos en el Modelo de Cuidado Integral-humanizado	Acción terapéutica propuesta en el Modelo de Cuidado Integral-humanizado	Intervenciones de enfermería (NIC)	Actividades
Acompañamiento	Vinculación afectiva	Apoyo emocional	Lecturas
		Potenciación de la autoestima	Discusión abierta
		Clarificación de valores	Autobiografía
		Terapia de grupo	Actividades recreativas/visitas guiadas
	Socialización grupal	Entrenamiento de la asertividad	Convivencia social
		Potenciación de la socialización	Juegos de mesa
		Aumentar los sistemas de apoyo	Fomento de las relaciones familiares y con amigas
		Modificación de la conducta: habilidades sociales	Actividad física
Orientación de salud	Educación para la salud	Apoyo al cuidador principal	Charlas educativas
		Mejorar el afrontamiento	Colorear/manualidades
		Disminución de la ansiedad	Actividades de relajación y meditación
	Seguimiento de la salud	Potencializar la autoconciencia	Reflexiones sobre su vida
		Apoyo emocional	Escucha activa
		Orientación de la realidad	Actividad física
		Entrenamiento de la memoria	Juegos de memoria
		Estimulación cognitiva	Juegos de memoria/juegos de mesa
		Disminución de la ansiedad	Musicoterapia/aromaterapia
Fe y esperanza	Afrontamiento de pérdidas	Asesoramiento	Charlas educativas
		Terapia familiar	Reflexiones sobre su vida
		Facilitar el perdón	Mapa cognitivo
		Apoyo en la toma de decisiones	Discusión abierta
	Grupo de confort espiritual	Dar esperanza	Terapia de reminiscencia
Diálogo terapéutico	Escucha activa	Apoyo a la familia	Sopa de letras
		Facilitar el duelo	Escribir cartas
		Estimulación de la integridad familiar	Fomento de las relaciones familiares y con amigas
	Foros de discusión	Cuidados por relevo	Musicoterapia
		Fomentar la implicación familiar	Charlas educativas
Amor	Permisión de expresión de sentimientos y emociones	Apoyo emocional	Terapia de reminiscencia
		Control del estado de ánimo	Musicoterapia/aromaterapia
	Afecto y empatía	Terapia de relajación	Colorear/manualidades
		Mejorar la autoconfianza	Actividad física/juegos de mesa

*Fuente: Valoración integral y diagnósticos de enfermería en adultas mayores del Valle de Toluca, 2024.*

**Cuadro 3:** Cuidados, acciones terapéuticas e intervenciones de enfermería en la esfera espiritual de las adultas mayores, Toluca, Estado de México, México

Cuidados propuestos en el Modelo de Cuidado Integral-humanizado	Acción terapéutica propuesta en el Modelo de Cuidado Integral-humanizado	Intervenciones de enfermería (NIC)	Actividades
Fe y esperanza	Afrontamiento de pérdidas	Apoyo espiritual	Reflexiones sobre su vida
		Modificación de sí mismo	Autoconocimiento/autobiografía
		Biblioterapia	Lectura de poemas o escritos literarios
		Dar esperanza	Incorporación de elementos de su cultura
		Facilitar la autorresponsabilidad	Fomento del autocuidado
		Presencia	Aceptación, empatía, confianza y apoyo
	Grupos de confort espiritual	Facilitar la práctica religiosa	Oraciones y agradecimientos
		Terapia de relajación	Aromaterapia
		Potenciación de las aptitudes para la vida diaria	Relaciones sanas
		Facilitar la meditación	Imaginación simple dirigida
		Apoyo espiritual	Reflexiones sobre su vida
		Intermediación cultural	Discusión abierta
Dialogo terapéutico	Foros de discusión	Ayuda con el autocuidado	Autocuidado
		Enseñanza individual	Charlas educativas
		Musicoterapia	Musicoterapia
		Apoyo en la toma de decisiones	Escucha activa
		Modificación de la conducta	Autoconocimiento
	Escucha activa	Escucha activa	Escucha activa
		Reestructuración cognitiva	Imaginación simple dirigida
		Imaginación simple dirigida	Historias
		Apoyo espiritual	Reflexiones sobre su vida
		Mejorar el afrontamiento	Fomento de las relaciones familiares y con amigas
		Disminución de la ansiedad	Terapias de relajación
		Dar esperanza	Incorporación de elementos de su cultura
		Apoyo emocional	Escucha activa
Acompañamiento	Vinculación afectiva	Facilitar el crecimiento espiritual	Reflexiones sobre su vida
		Mantenimiento de procesos Familiares	Discusión abierta
		Asesoramiento	Charlas educativas
	Socialización grupal	Facilitar el crecimiento espiritual	Reflexiones sobre su vida
	Grupos de apoyo mutuo	Aromaterapia	Aromaterapia

*Fuente: valoración integral y diagnósticos de enfermería en adultas mayores del Valle de Toluca, 2024.*

## RESULTADOS

Una vez finalizada esta fase de intervención, se procedió a la evaluación del servicio en dos etapas: por un lado, se reaplicaron los instrumentos de evaluación para determinar los efectos o resultados sobre la salud de las mujeres. Los resultados se analizaron de forma cuantitativa, con el *pre* y *post-test* de las escalas de valoración geriátrica aplicadas a las adultas mayores al inicio y al final del periodo en que se brindó la consulta de enfermería.

Por otro lado, se aplicó la Escala de Cuidado Profesional de Kristen Swanson, con la cual se evalúo la percepción de las mujeres mayores sobre el cuidado de enfermería recibido. Finalmente, también se presentan algunos comentarios de las entrevistas no estructuradas realizadas durante el periodo de intervención.

### Pre y post-test de la valoración geriátrica

Con la finalidad de complementar la valoración integral de enfermería (por patrones funcionales), se aplicaron escalas validadas de valoración geriátrica en las esferas humanas antes detalladas.

Para la esfera físico-funcional, se realizó la aplicación de la Escala de Valoración Nutricional, en donde se obtuvo una media de 6.3 puntos en el *pre-test*, lo que se interpretó como riesgo nutricional alto en las adultas mayores. En el *post-test*, se obtuvo una media de 4.8 puntos, interpretado como riesgo nutricional moderado. Cabe mencionar que, dentro de las intervenciones realizadas, se promovió e incentivó la ingesta de alimentos más saludables.

También en esta esfera, se ministró la Escala de Tinetti (marcha y equilibrio), mediante la cual no se observaron cambios significativos en la evaluación post-intervención, manteniendo un promedio entre 24.6 y 25 puntos que se interpreta como un riesgo bajo.

En lo que respecta a la esfera psico-social, el Cuestionario Apgar de la Familia nos apoyó a verificar la funcionalidad familiar de las adultas mayores participantes, obteniendo en el *pre-test* una puntuación media de 12.3, que se interpreta como disfunción moderada, y en el *post-test*, una media de 14.3 puntos, interpretada como una disfunción leve. Destacando que las intervenciones de enfermería enfocadas en la resolución de conflictos y perdón, pudieron haber influido para cambiar la percepción de las relaciones familiares de las adultas mayores.

La Escala de Recursos Sociales se utilizó con la finalidad de evaluar la calidad de las redes de apoyo de las adultas mayores. Se pudo observar que cada adulta mayor tiene características específicas con base en sus roles sociales y el de sus hijos y pareja; sin embargo, todas tienen deterioro de sus recursos sociales. En el *pre-test*, se identificó que una de ellas tiene riesgo social grave, dos con deterioro social moderado y tres con deterioro social leve. En el *post-test*, no hubo cambios considerables, con una adulta mayor con riesgo social grave, una con deterioro social moderado, dos con deterioro social leve y dos con buenos recursos sociales.

Mediante el Mini-Examen del Estado Mental, se pudo identificar que durante el periodo de encuestas *pre* y *post-test*, las adultas mayores no presentaron datos evidentes de deterioro cognitivo, manteniéndose en un promedio de 29.8 puntos en el pre y de 32.1 en el post, lo que según esta escala se interpreta como dentro de la normalidad. Hay una pequeña variación hacia la mejora, que pudo ser por las actividades cognitivas y de memoria que se trabajaron con las adultas mayores, incluyendo lectura y escritura.

Además, se aplicó el Inventario de Ansiedad de Beck, donde al inicio las adultas mayores tuvieron una media de 6.8 puntos. que indica una ansiedad normal, sin embargo, en el post-test, obtuvieron una puntuación promedio de 13.8, indicando ansiedad leve.

Con la Escala de Depresión Geriátrica (Brink y Yesavage), se volvió a comprobar que las mujeres adultas mayores que son funcionales e independientes no tienen depresión. Las medias pre y post-test fluctuaron entre los 3.5 y 3.2 puntos, interpretándose como un estado normal.

Finalmente, para la esfera espiritual, se aplicó la Escala Trifactorial de Espiritualidad en personas mayores mexicanas, en la que las mujeres al inicio de la valoración obtuvieron un promedio de 134 puntos y. al final de la intervención, de 138.6 puntos. Esto quiere decir que las adultas mayores tienen una espiritualidad buena.

### Escala de Cuidado Profesional de Kristen Swanson en la atención recibida en la consulta de enfermería

Como parte de la evaluación del cuidado de enfermería, es importante conocer la percepción de las adultas mayores sobre la actitud, humanismo y valores que el personal demostró en la consulta de enfermería. La puntuación promedio obtenida mediante la Escala de Cuidado Profesional de Kristen Swanson fue de 58.3, lo cual, según la autora de la escala, indica que el cuidado profesional es considerado excelente. Implica estar completamente presente, con la curación de amor que fluye. La personalidad se reconoce mutuamente; la atención se negocia; y la intimidad profesional se produce. Esta relación favorece la libertad espiritual. Este resultado era el esperado con la implementación del Modelo de Cuidado Integral-Humanizado para adultas mayores.

### Comentarios

Durante las entrevistas abiertas realizadas en la consulta de enfermería, se observaba que las adultas mayores disfrutaban de la compañía y de ser escuchadas, les gustaban las plantas y sus mascotas, con quienes conversaban, y anhelaban la paz y la tranquilidad. Eran gentiles y participativas en las actividades, inclusive compartían con los investigadores comida y actividades de su vida diaria. Aquí algunos comentarios de la sesión final:

*Es triste saber que ya no los vamos a volver a ver.* (AM1)

*Muchas gracias por tanto cariño y afecto. Muchas felicidades por tantas intenciones.* (AM2)

*¿Qué, van a sanar nada más las heridas exteriores? Tienen que sanar las heridas interiores de esos pacientes.* (AM3)

Además, las adultas mayores realizaron un diario de experiencias durante el periodo de intervención, destacando los siguientes fragmentos:

*Mi camino con las enfermeras Laura, Gela, Dani y Diego. Para tener una etapa saludable en mi tercera juventud, he relatado como he transcurrido mi vida desde la infancia hasta llegar a donde estoy ahora, y es en esta etapa donde, a veces, ha sido con nostalgia. Pienso que debemos acostumbrarnos a vivir nuestra propia vida, a cuidarnos, porque nadie más que nosotros sabemos lo que nos duele; hay que valernos por sí mismos mientras podamos.* (AM1)

*Mi llegada a la tercera edad ha sido como una tarde de verano, una vida de recuerdos maravillosos, experiencias, de reflexiones, de todo lo que pude aprender, conocer, reconocer el gran amor de Dios, de todo lo maravilloso que me permitió tener: a mis tres hijos, que debo prepararme para la vejez, a tener una vida más saludable, a dedicarme a sí misma, pues como ya no tengo un empleo, necesito ayuda para no deprimirme, para darme y dedicarme a mí, para conocer lugares, prepararme para una vejez más tranquila y saludable y acercarme a Dios, que él siempre está conmigo, y ser cada día más independiente.* (AM2)

## DISCUSIÓN

Los resultados obtenidos demuestran la efectividad del Modelo de Cuidado Integral-humanizado en las dimensiones que impactan la salud y bienestar de las adultas mayores.

Estos resultados coinciden con Yushan Yu^(12)^, quienes, a través de una revisión sistemática, concluyen sobre la importancia de adaptar los servicios domiciliarios acorde a las necesidades los adultos mayores, así como seguir investigando para proporcionar mejores servicios comunitarios y domiciliarios. Para la esfera físico-funcional, mediante la estrategia de orientación en salud, se promovió el autocuidado con el consumo de alimentos saludables, lo que coincide con la evidencia presentada en revisiones sistemáticas y estudios documentales^(13,14)^. Sumado a ello, se resalta la participación grupal de las adultas mayores, lo cual marca una diferencia en la mejora presentada en los resultados correspondientes a esta esfera y que también tienen relación con lo presentado en la revisión sistemática de Teggart^(15)^, al mismo tiempo que enfatiza seguir investigando el estado nutricional en los adultos mayores.

Los resultados obtenidos en la esfera psico-social se empatan con los encontrados por Sánchez-Coronel^(16)^, quien desde el contexto mexicano reconoce que la falta de estrategias en el abordaje de la población adulta mayor y las dinámicas familiares limita el campo de acción, lo cual es atendido en la presente investigación, evidenciándose como un conjunto de intervenciones estructuradas y previamente comprobadas por la taxonomía NIC, que mejoraron las dinámicas sociales y emocionales de las participantes. En esta esfera, también se demuestra como la influencia de trabajo en grupo, en conjunto con las intervenciones individualizadas en la resolución de conflictos y perdón, puede mejorar la dinámica familiar y el autocuidado espiritual^(17)^. Los datos obtenidos dejan en evidencia que las adultas mayores que participaron, al no tener un cuidado directo dentro de la dinámica familiar, se suman a la necesidad planteada por la Organización Mundial de la Salud^(18)^ de las ciudades y comunidades amigables con las personas mayores, las cuales fomentan un envejecimiento saludable al optimizar recursos de salud y seguridad.

Respecto a los aspectos cognitivos, la literatura coincide que las actividades de estimulación cognitiva regular, como las implementadas en este estudio (lectura, escritura, juegos de memoria), pueden mantener y mejorar las funciones mentales^(18-20)^. Un hallazgo de los resultados es el aumento del nivel de ansiedad de normal a leve, lo cual es secundario a la aplicación de la intervención de reminiscencia, si bien no está claramente demostrada como una intervención efectiva en el tratamiento de la ansiedad, deterioro cognitivo y la depresión^(21)^; cuando menos documentado, produce el efecto de la ciencia, recordando preocupaciones existentes. Este hecho sugiere que se debe seguir trabajando con estas intervenciones para paliar estas sensaciones^(6)^ y promover un proceso de confrontación.

Ante el estado de salud de las adultas mayores y las evidentes mejoras mostradas en los resultados, se considera dentro del Modelo de Cuidado Integral-humanizado la dimensión espiritual y percepción del cuidado de enfermería recibido como parte de la base de una salud holística, ya que el estado espiritual permite potenciar los beneficios de las intervenciones antes descritas. Por ello, Soto^(22)^, quien hace un análisis construyendo un estado del arte basado en la literatura, se ha convertido en un elemento de conocimiento para la enfermería y en específico, confirmando la utilidad de éste en el cuidado de la vida de los adultos mayores^(7,23)^.

Estas consideraciones permiten amalgamar una serie de intervenciones individualizadas, lo que se ve reflejado en la evaluación del cuidado profesional, validando la efectividad del Modelo de Cuidado Integral-humanizado. Además, se considera que dicha satisfacción está vinculada con la atención a domicilio, como lo reportan estudios de Brasil y España^(24-27)^, que relacionan la satisfacción con el cuidado a la atención personalizada y holística.

La efectividad de las intervenciones espirituales se refleja en testimonios como “desarrollar y mantener a edades avanzadas la capacidad funcional que hace posible el bienestar”, que demuestran una conexión entre el bienestar espiritual y la funcionalidad.

### Limitaciones del estudio

Se puede considerar que la muestra fue pequeña, debido a la dificultad para integrar un grupo de adultas mayores que cumplieran con los requisitos del estudio y que, sobre todo, contaran con la disponibilidad y el tiempo para acudir a la consulta de enfermería, ya que, en México, la mayoría de mujeres mayores fungen el rol de cuidadoras de los nietos y del hogar, dificultando la asistencia de las adultas mayores a actividades o intervenciones para el cuidado de sí mismas.

### Contribuciones para el área de enfermería

Como parte de las contribuciones especificas del Modelo de Cuidado Integral-humanizado, se encuentra su aplicación a la consulta de enfermería en el área geriátrica, mostrando la autonomía y la eficiencia de los cuidados e intervenciones con una base espiritual, y las mejoras en las esferas físicas y psicosociales. Además, se amplían la evidencia científica y la integración de la teoría de Kristen Swanson con la práctica comunitaria de enfermería.

## CONSIDERACIONES FINALES

Los resultados de las intervenciones de enfermería fueron positivos en el bienestar de las adultas mayores. En lo referente a la funcionalidad, mediante los ejercicios y actividad física implementados como parte de las intervenciones, las mujeres sintieron menos dolor en sus articulaciones y más fuerza en sus piernas. En lo psicosocial, a medida que se lograba la confianza de las adultas mayores, se verbalizaron algunos problemas o actitudes de sus familiares que las hacían sentirse mal. Sin embargo, se notaba que estaban más contentas por acudir a realizar las actividades propuestas por el grupo de investigación y se forjaron lazos de amistad entre las adultas mayores, construyendo su propia red de apoyo. Se permitió la expresión de pensamientos y actividades espirituales, que les brindaron más tranquilidad y paz en su vida.

Se sugiere replicar la metodología y el modelo en poblaciones más amplias de adultas mayores para comparar los resultados con los de este estudio.

## Data Availability

Los datos de investigación están disponibles en el repositorio: https://doi.org/10.48331/scielodata.JHVJAT.
